# Effects of G-CSF on hPDLSC proliferation and osteogenic differentiation in the LPS-induced inflammatory microenvironment

**DOI:** 10.1186/s12903-023-03040-9

**Published:** 2023-06-26

**Authors:** Hui Yu, Pengcheng Wang, Haibin Lu, Jiurong Guan, Fang Yao, Tianyi Zhang, Qiuxu Wang, Zuomin Wang

**Affiliations:** 1 Department of Stomatology, Affiliated Zhongshan Hospital of Dalian University, 6th Jiefang Street, Dalian, Liaoning China; 2 Department of Stomatology, Beijing Chao-Yang Hospital, Capital Medical University, 8th Gongti South Road, Beijing, China; 3 Shanxi Medical University, 382th WuyiRoad, Xinghualing Distrct, Taiyuan, Shanxi China

**Keywords:** Periodontitis, G-CSF, hPDLSCs, Osteogenic differentiation

## Abstract

**Background:**

Periodontitis is a chronic infectious disease of periodontal support tissue caused by microorganisms in dental plaque, which causes alveolar bone resorption and tooth loss. Periodontitis treatment goals include prevention of alveolar bone resorption and promotion of periodontal regeneration. We previously found that granulocyte colony-stimulating factor (G-CSF) was involved in periodontitis-related alveolar bone resorption through induction of an immune response and subsequent destruction of periodontal tissue. However, the mechanisms underlying the effects of G-CSF on abnormal bone remodeling have not yet been fully elucidated. Human periodontal ligament stem cells (hPDLSCs) are major modulators of osteogenic differentiation in periodontal tissues. Thus, the aim of this study was to investigated whether G-CSF acts effects on hPDLSC proliferation and osteogenic differentiation, as well as periodontal tissue repair.

**Methods:**

hPDLSCs were cultured and identified by short tandem repeat analysis. The expression patterns and locations of G-CSF receptor (G-CSFR) on hPDLSCs were detected by immunofluorescence analysis. The effects of G-CSF on hPDLSCs in a lipopolysaccharide (LPS)-induced inflammatory microenvironment were investigated. Specifically, Cell-Counting Kit 8 (CCK8) and Alizarin red staining were used to examine hPDLSC proliferation and osteogenic differentiation; reverse transcription-polymerase chain reaction was performed to detect the expression patterns of osteogenesis-related genes (alkaline phosphatase [ALP], runt-related transcription factor 2 [Runx2], and osteocalcin [OCN]) in hPDLSCs; and Western blotting was used to detect the expression patterns of phosphatidylinositol 3-kinase (PI3K) and protein kinase B (Akt) of PI3K/Akt signaling pathway.

**Results:**

hPDLSCs exhibited a typical spindle-shaped morphology and good clonogenic ability. G-CSFR was mostly localized on the cell surface membrane. Analyses showed that G-CSF inhibited hPDLSC proliferation. Also, in the LPS-induced inflammatory microenvironment, G-CSF inhibited hPDLSC osteogenic differentiation and reduced the expression levels of osteogenesis-related genes. G-CSF increased the protein expression levels of hPDLSC pathway components p-PI3K and p-Akt.

**Conclusions:**

We found that G-CSFR was expressed on hPDLSCs. Furthermore, G-CSF inhibited hPDLSC osteogenic differentiation in vitro in the LPS-induced inflammatory microenvironment.

**Supplementary Information:**

The online version contains supplementary material available at 10.1186/s12903-023-03040-9.

## Introduction

Periodontitis constitutes chronic inflammation of periodontal support tissue caused by microorganisms in dental plaque; it can lead to periodontal tissue destruction and tooth loss. Importantly, tooth loss causes esthetic and functional problems; it also reduces quality of life. Periodontitis has been associated with the presence of systemic diseases, including Alzheimer’s disease [1], coronary heart disease [2], and both lung and pancreatic cancers [3]. The routine treatment of periodontitis involves the removal of dental plaque by supragingival cleansing and subgingival scaling to prevent the progression of periodontitis lesions. However, the damage to periodontal tissue (e.g., alveolar bone) is difficult to reverse. Some researchers have proposed that tissue destruction in periodontitis is not always directly caused by plaque microorganisms; it may be indirectly caused by the host response to infected microorganisms and their toxic products [4]. Thus, periodontitis is a local manifestation of the systemic immune response in the periodontal setting.

Immune factors are involved in the periodontal tissue repair, such as high transforming growth factor-β1 production might be a protective factor for periodontitis [5], vitamin D levels were positively correlated with the number of teeth and negatively with C-reactive protein and all periodontal parameters [6]. However, various inflammatory mediators that participate in bone loss and collagen degradation in periodontitis; these include interleukin (IL) family such as IL-1, IL-6 [7], tumor necrosis factor-α, matrix metalloproteinases [8], and granulocyte colony-stimulating factor (G-CSF) [9]. Therefore, a thorough exploration of the immunological pathogenesis of periodontitis and search for new treatment options will greatly promote the prevention of periodontitis.

G-CSF is a neutrophil-specific myeloid cytokine and an important component of systemic immunity. During local infection and inflammation, various cellular components (e.g., endothelial cells, fibroblasts, and macrophages) can synthesize and secrete G-CSF [10]. Additionally, periodontitis is an infectious disease that is characterized by high expression levels of G-CSF. In vitro stimulation with *Porphyromonas gingivalis* was reported to cause cultured macrophages to synthesize and secrete G-CSF [11]. And stimulation with heat-inactivated *P. gingivalis* was found to increase G-CSF secretion by macrophages [12]. Additionally, periodontal pathogen-derived lipopolysaccharide (LPS) can elicit high expression levels of G-CSF in gingival epithelial cells and fibroblasts [9, 13]. In clinical studies, transcriptomic sequencing analysis of patients with periodontitis revealed that the G-CSF gene exhibited significantly altered expression among the 400 genes with increased levels in inflammatory periodontal tissue [14]. Furthermore, upon stimulation with subgingival plaque or LPS, peripheral blood cells from periodontitis patients produce higher levels of G-CSF [15] than do such cells from healthy individuals. The above studies indicated that periodontitis with increased expression of G-CSF may be one of the important contributing factors of inflammatory periodontal tissue.

Experimental analyses have shown that G-CSF transgenic mice exhibited severe osteoporosis, with a significantly elevated number of osteoclasts on the bone surface and significant enhancement of bone resorption [16]. Clinical studies have demonstrated that patients receiving long-term G-CSF treatment have a significantly increased risk of osteoporosis; they also exhibited significant reductions of bone mineral density [17]. Furthermore, exogenous G-CSF significantly reduced the number of osteoblasts on the surface of bone tissue; it also significantly reduced the gene expression level of osteocalcin (OCN) [18]. Clinical studies have revealed significant reductions in the numbers of osteoblasts, as well as the expression levels of osteoprotegerin and OCN, in patients receiving G-CSF treatment [19]. In contrast, G-CSF knockout mice exhibited significant bone structure improvement compared with wild-type mice; these improvements involved the percentage and thickness of trabecular bone, as well as the area and thickness of bone trabecula [20]. In our previous study, we confirmed these findings in periodontal tissue in an animal model [21]: G-CSF expression levels were significantly upregulated in both serum and gingival epithelial cells. Moreover, anti-G-CSF antibody administration was sufficient to alleviate alveolar bone resorption.The above studies indicated that G-CSF may be an essential component of the immune response that contributed to bone loss in periodontitis. However, the mechanisms underlying the effects of G-CSF on abnormal bone remodeling have not yet been fully elucidated.

The most serious damage in periodontitis is caused by alveolar bone resorption. While alveolar bone exhibits the greatest metabolic and remodeling activity within the human bone system [22]. Human periodontal ligament stem cells (hPDLSCs) have important roles in the modulation of osteogenic differentiation in periodontal tissues, which offer an ideal cellular source for the promotion of periodontal tissue repair and regeneration [23]. Additionally, hPDLSCs can differentiate into osteoblasts and similar cells (e.g., adipocytes and chondrogenic cells) under specific culture conditions [24]. G-CSF is known to block proliferation and osteogenic differentiation in hematopoietic stem cells, induce apoptosis in osteoblasts, and reduce new bone formation [25]. The use of G-CSF induced mobilization for isolation of dental pulp stem cells with high regenerative potentialen, were riched for CD105, C-X-C chemokine receptor type 4 and G-CSFR positive cells [26].Thus far, the expression patterns of G-CSFR in periodontal tissue cells have not been characterized. While, whether G-CSF acts effects on hPDLSC, as well as periodontal tissue repair, have not yet been identified.

In light of these results, the primary aim of this study was focused on whether G-CSF acts through effects on hPDLSC proliferation and osteogenic differentiation in a LPS-induced inflammatory microenvironment. To determine the roles of G-CSF on hPDLSC, we investigated the localization and expression of G-CSFR, as well as the osteogenic differentiation and expression patterns of osteogenesis-related genes, also protein expression levels of PI3K/Akt signaling pathway. With the aim of providing a theoretical basis for periodontal tissue repair and regeneration in periodontitis.

## Materials and methods

### Cells and cell culture

hPDLSCs (HUM-iCELL-m002; iCell Bioscience Inc., Shanghai, China) were cultured at a density of 1⋅10^4^ cells/well in Dulbecco’s modified Eagle medium, in an atmosphere of 5% CO_2_ at 37 °C.

### Immunofluorescence staining

Immunofluorescence staining was performed in accordance with standard protocols. Briefly, cells on slides were air-dried, then fixed in 4% paraformaldehyde overnight. Subsequently, endogenous peroxidases were inactivated by incubation in 3% H_2_O_2_–methanol at room temperature for 10 min. Slides were then blocked with goat serum sealant (KGSP03, Keygen, Jiangsu, China) at room temperature for 20 min. Immunofluorescence staining was performed using a primary antibody to G-CSF receptor (G-CSFR; 1:100 dilution, ab126167, Abcam, Cambridge, MA, USA) by incubation in a humidified environment for 2 h at 37℃. Secondary antibody detection was performed with goat anti-rabbit fluorescein isothiocyanate (1:100 dilution, 111-095-003, Jackson ImmunoResearch, West Grove, PA, USA) by incubation in darkness at 37℃ for 1 h. Slides were sealed by 5 min of incubation at room temperature with Vectashield mounting medium plus 4’,6-diamidino-2-phenylindole (DAPI) (KGA215, Keygen). Imaging was performed with a confocal microscope (BX43, Olympus, Tokyo, Japan).

### Cell-counting kit 8 (CCK-8) assay

hPDLSCs at passages 3 and 4 were used for experiments. Cells were digested, counted, and suspended at a concentration of 5⋅10^4^ cells/mL. One hundred microliters of cell suspension were added to each well of a 96-well cell culture plate, then incubated at 37 °C for 24 h. The cells were divided into three experimental groups. All groups were treated with 100 µl of media containing the components listed below, then incubated for 48 or 72 h at 37 °C. Cells in the G-CSF group were treated with G-CSF (AP74769, SAB, USA) at a concentration of 0.0001, 0.001, 0.01, 0.05, 0.1, 1, 10, or 20 µg/mL. Cells in the LPS group were treated with LPS (L2630, Sigma-Aldrich, USA) at a concentration of 0.0001, 0.001, 0.01, 0.1, 1, 10, 20, 50, or 100 µg/mL. Cells in the G-CSF + LPS group were treated with an optimal concentration and time according to the results of the above concentration gradients. After treatment, 10 µl of CCK-8 solution (KGA317, Keygen) were added to each well and the cells were incubated at 37 °C for 2 h. Cell inhibition ratios were determined by the reading the absorbance at 450 nm using a microplate reader.

### Chromatin red

hPDLSCs were seeded in six-well plates at a density of 2⋅10^4^ mL/well. When the cells covered approximately 90% of the well bottom, the medium was changed to osteogenic induction medium (iCell-MSCYD-002, iCell Bioscience Inc.) to induce osteogenic differentiation. The medium was changed every 3 days thereafter. Cell cultures were terminated at day 21, then fixed in 4% paraformaldehyde for 15 min and stained with 0.1% alizarin red for 30 min. They were imaged by a light microscope (BX53, Olympus). The images were analyzed with Image-Pro Plus 6.0 software (Media Cybernetics Inc., Bethesda, MD, USA) to determine the ratio of alizarin red-stained cells to the total area.

### Reverse transcription-polymerase chain reaction (RT-PCR)

Total RNA was extracted using TRIzol (15596-026, Invitrogen, USA). Two micrograms of total RNA were collected from each group, then used to generate cDNA via reverse transcription in a total volume of 20 µL. Next, real-time quantitative PCR was performed using One Step TB Green™ PrimeScript™ RT-PCR Kit II (SYBR Green) (RR086B, TaKaRa, Japan). The conditions of denaturation, annealing and extension were as follows: initial denaturation at 95 °C for 5 min, followed by 40 cycles of 95 °C for 15 s, 60 °C for 20 s, and 72 °C for 40 s. Relative gene expression levels were analyzed by the 2^−ΔΔct^ method and standardized according to the level of glyceraldehyde-3-phosphate dehydrogenase (GAPDH). The following primers were used in the experiment:

GAPDH forward, 5’-AGATCATCAGCAATGCCTCCT-3’ and GAPDH reverse, 5’-TGAGTCCTTCCACGATACCAA-3’;

alkaline phosphatase (ALP) forward, 5’-ACTCTCCGAGATGGTGGTGGTG-3’ and ALP reverse, 5’-CCGTGGTCAATTCTGCCTCCTT-3’;

RUNX2 forward 5’-CCCAGGCAGTTCCCAAGCATTT-3’ and RUNX2 reverse, 5’-GGTAGTGAGTGGTGGCGGACAT-3’;

OCN forward 5’-GGCAGCGAGGTAGTGAAGAGAC-3’ and OCN reverse 5’-GGTCAGCCAACTCGTCACAGTC-3’.

### Western blotting analysis

Cellular proteins were extracted using a whole-protein extraction kit (KGP250, Keygen). Total protein was measured using a micro bicinchoninic acid protein determination kit (KGA902, Keygen). Thirty micrograms of protein were separated by 10% sodium dodecyl sulfate–polyacrylamide gel electrophoresis. Then electroblotted onto polyvinylidene difluoride membranes. The membranes were blocked with 5% milk in Tris-buffered saline plus Tween at room temperature for 2 h, then incubated at 4 °C overnight with primary antibody (protein kinase B [Akt], 1:1000 dilution, ab179463, Abcam; p-Akt, 1:5000 dilution, ab81283, Abcam; phosphatidylinositol 3-kinase [PI3K], 1:1000 dilution, ab191606, Abcam; p-PI3K, 1:1000 dilution, ab182651, Abcam) in 1% milk in Tris-buffered saline plus Tween. Finally, they were incubated for 2 h with goat anti-mouse IgG horseradish peroxidase-conjugated secondary antibody (KGAA37, Keygen). Protein bands were detected using enhanced chemiluminescent reagents (KGP116, Keygen).

### Statistical analysis

IBM SPSS Statistics software, version 22.0 (IBM Corp., Armonk, NY, USA) was used for statistical analysis. All quantitative data are presented as means ± standard deviations. Statistical analyses were performed using one-way ANOVA between groups in Post hoc multiple comparisons with LSD test to identify statistically significant differences. Values of *P* < 0.05 were considered statistically significant.

## Results

### Biological properties of hPDLSCs

Morphology of hPDLSCs—Microscopy revealed that hPDLSCs were circular or polyangular in shape, with nuclei clustered at the center and cytoplasmic processes that were spindle-shaped in a radial pattern (supplementary file Fig. S1); the cells also showed good clonogenic ability. Thus, hPDLSCs exhibited typical fibroblast-like morphology, with the characteristics of adult stem cells. Immunofluorescence identification: STRO-1 immunofluorescence was detected, indicating cell purity of > 90% (supplementary file Fig. S2). Identification of short tandem repeats—Genotyping results and typing profiles from the short tandem repeats and amelogenin loci of hPDLSCs indicated clear genomic DNA amplification of hPDLSCs and good typing results (supplementary file Fig. S3).

### G-CSFR expression and localization in hPDLSCs

**Fig. 1:**
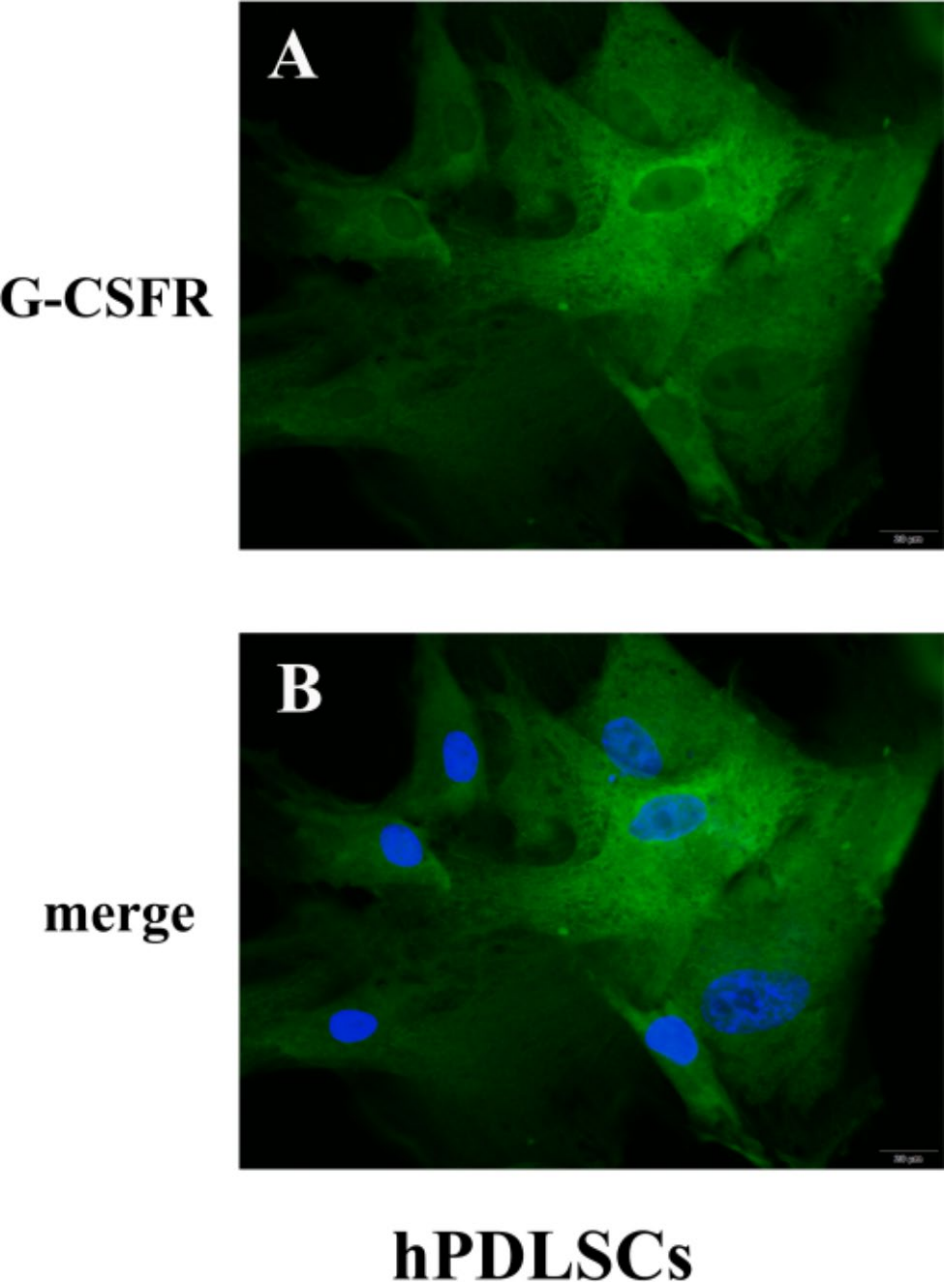
Immunofluorescence analysis of G-CSFR expression and localization in hPDLSCs. hPDLSC membranes exhibited green fluorescence (**A**), while the nuclei exhibited blue fluorescence (**B**). Bar: 20 μm

To evaluate G-CSFR expression and localization in hPDLSCs, we performed immunofluorescence analysis. Our findings confirmed that G-CSFR was expressed in hPDLSCs. Most G-CSFR protein expression in hPDLSCs was localized to the membrane and within the cytoplasm (Fig. 1A); it was distinct from the nuclei (Fig. 1B).

### Effect of G-CSF on hPDLSC proliferation in the LPS-induced inflammatory microenvironment

#### Effect of G-CSF on hPDLSC proliferation

**Fig. 2:**
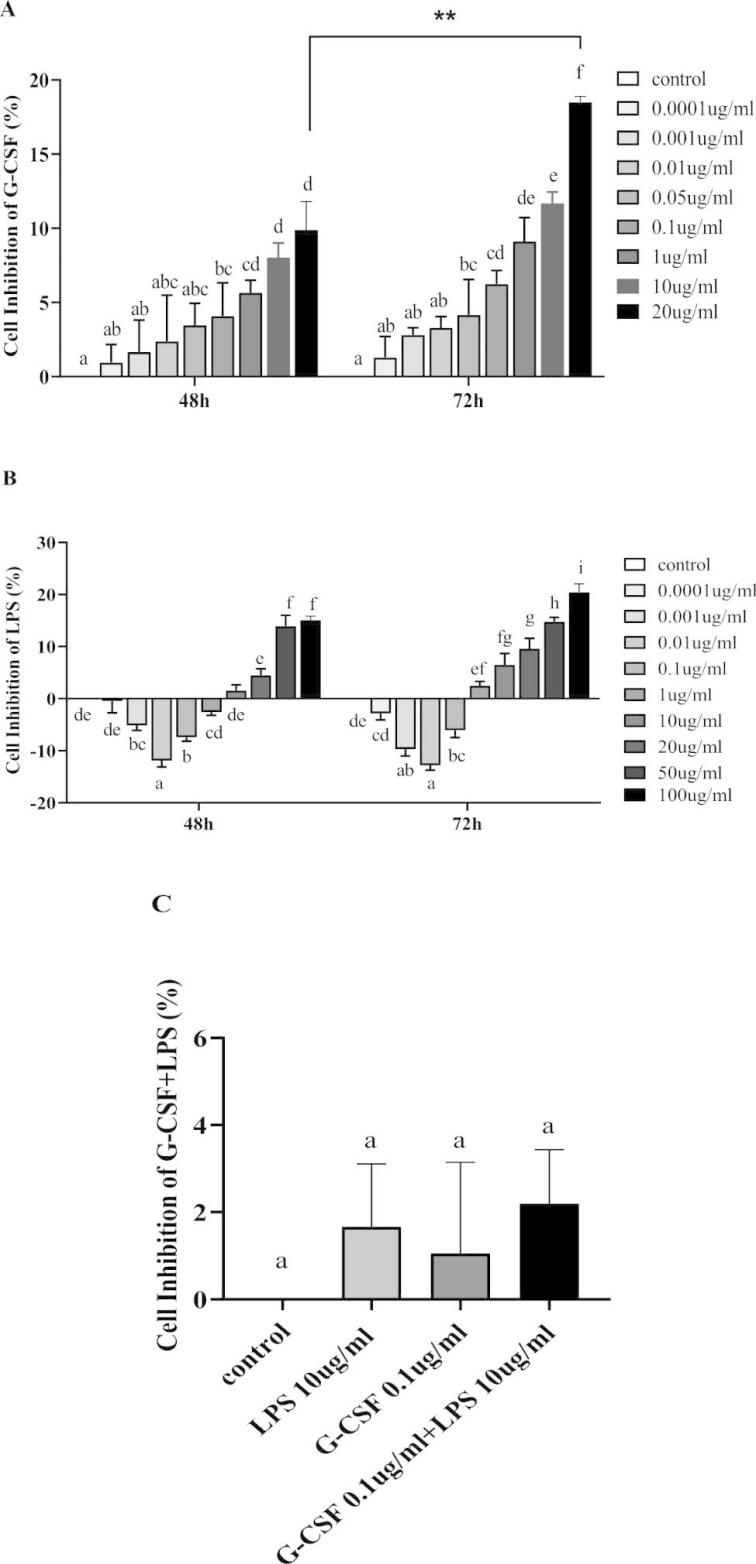
CCK-8 was used to examine the effect of G-CSF on hPDLSC proliferation in the inflammatory microenvironment. (**A**) Effect of G-CSF on hPDLSC proliferation. (**B**) Effect of LPS on hPDLSC proliferation. (**C**) Effect of G-CSF on hPDLSC proliferation in the LPS-induced inflammatory microenvironment. Pairwise comparisons were performed between different concentrations at the same time point; significant differences (*P* < 0.05) are indicated by different letters, while similar letters indicate no difference

Compared with the control group, an increasing concentration of G-CSF (0.0001–20 µg/mL) inhibited hPDLSC proliferation over time; the inhibitory effect gradually increased with the concentration of G-CSF. When the concentration of G-CSF was ≥ 1 µg/mL and hPDLSCs were stimulated for 48 h, the inhibition of hPDLSC proliferation significantly increased with an increasing concentration of G-CSF (i.e., among 1, 10, and 20 µg/mL; *P* < 0.05, Fig. 2A). When the concentration of G-CSF was ≥ 0.1 µg/mL and hPDLSCs were stimulated for 72 h, the inhibition of hPDLSC proliferation significantly increased with an increasing concentration of G-CSF (i.e., among 0.1, 1, 10, and 20 µg/mL; *P* < 0.05). Thus, the moderate cytoinhibitory concentration of 0.1 µg/mL G-CSF for 48 h was used in subsequent experiments.

#### Effect of LPS on hPDLSC proliferation

Compared with the control group, when the concentration of LPS was ≤ 1 µg/mL (0.0001–1 µg/mL) and hPDLSCs were stimulated for 48 h, the promotion of hPDLSC proliferation significantly increased with a decreasing concentration of LPS (*P* < 0.05, Fig. 2B). Furthermore, when the concentration of LPS was ≥ 10 µg/mL (10–100 µg/mL), the inhibition of hPDLSC proliferation significantly increased with an increasing concentration of LPS (*P* < 0.05). When hPDLSCs were stimulated with LPS for 72 h, an LPS concentration of ≤ 0.01 µg/mL significantly promoted hPDLSC proliferation (*P* < 0.05). When the concentration of LPS was ≥ 1 µg/mL, the inhibition of hPDLSC proliferation significantly differed. Therefore, the moderate cytoinhibitory concentration of 10 µg/mL LPS for 48 h was used in subsequent experiments.

Effect of G-CSF on hPDLSC proliferation in the LPS-induced inflammatory microenvironment.

Compared with the control group, the G-CSF, LPS, and G-CSF + LPS groups all exhibited inhibition of hPDLSC proliferation. Although the G-CSF treatment tended to enhance the inhibition of hPDLSC proliferation in the LPS-induced inflammatory microenvironment, this enhancement was not statistically significant (*P* > 0.05, Fig. 2C).

### Effects of G-CSF on hPDLSC osteogenic differentiation capacity in the LPS-induced inflammatory microenvironment

**Fig. 3:**
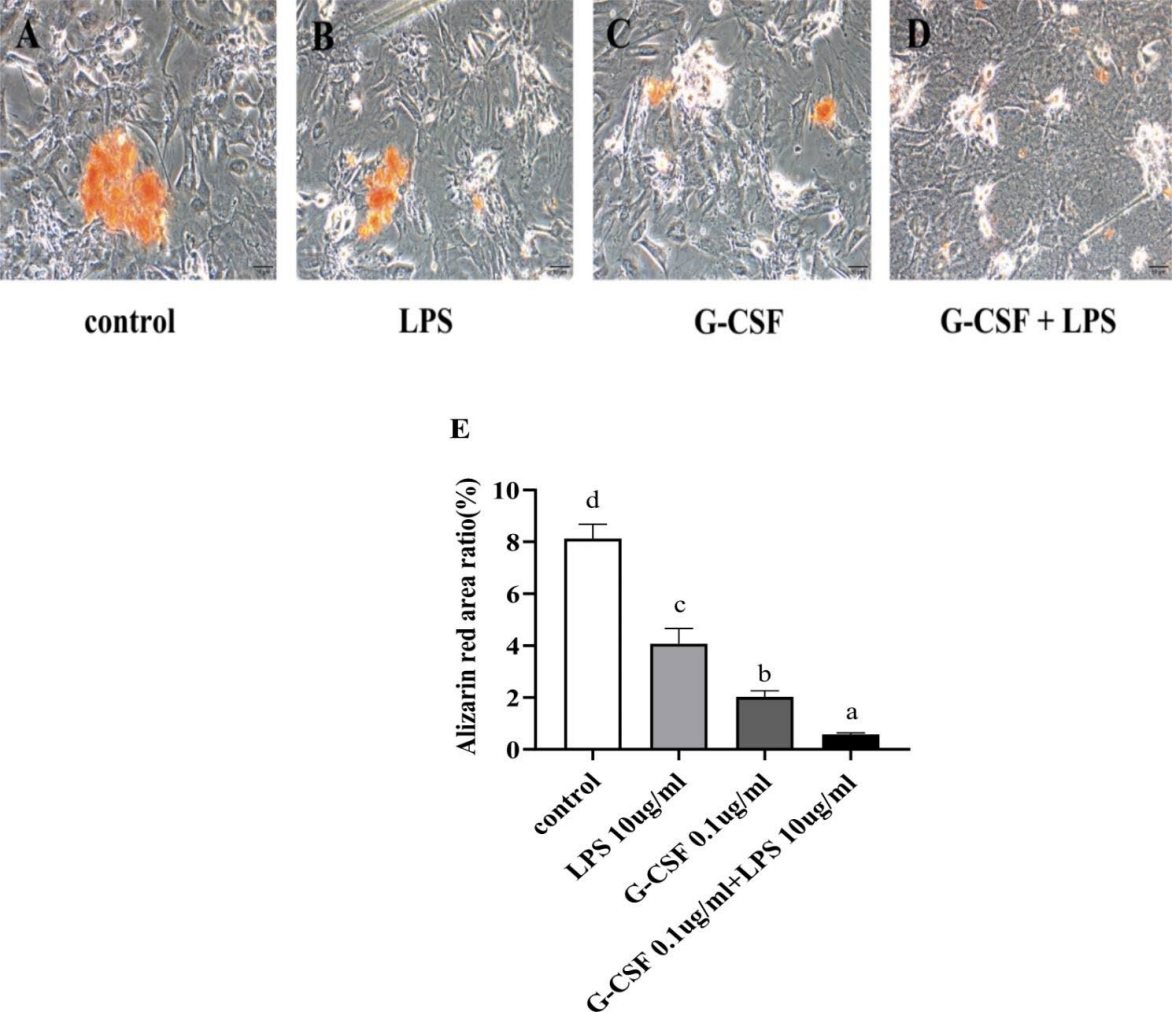
Alizarin-red staining assessment of G-CSF effects on hPDLSC osteogenic differentiation capacity in the LPS-induced inflammatory microenvironment. (**A**) Large areas of mineralized nodules (orange color) were visible in the control group. (**B**) There were fewer mineralized nodules in the LPS group (**C**) and in the G-CSF group. (**D**) The mineralized nodules were punctate in the G-CSF + LPS group. Bar: 50 μm. (**E**) Pairwise comparisons were performed between groups; significant differences (*P* < 0.05) are indicated by different letters, while similar letters indicate no difference

After 21 days of osteogenesis induction, alizarin-red staining revealed large areas of orange-red calcium nodules in the control group (Fig. 3A). The areas with mineralized nodules were smaller in the LPS group than in the control group (Fig. 3B); the sizes of such areas were greatly decreased in the G-CSF group (Fig. 3C). However, mineralized nodules were nearly absent or punctate in the G-CSF + LPS group (Fig. 3D). The differences between groups were statistically significant (Fig. 3E).

### Effect of G-CSF on the expression levels of hPDLSC-associated osteogenic markers in the LPS-induced inflammatory microenvironment

**Fig. 4:**
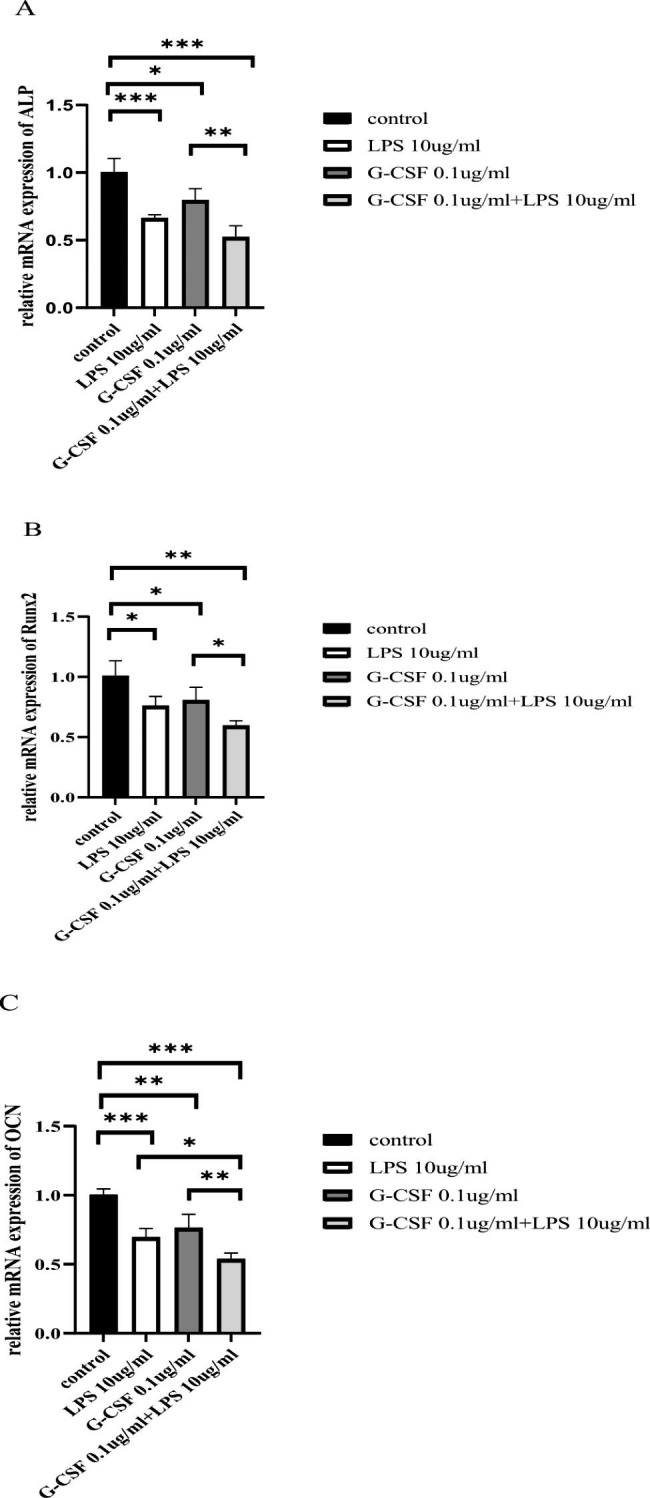
RT-PCR data showing effect of G-CSF on hPDLSC-associated osteogenic marker levels in the LPS-induced inflammatory microenvironment. (**A**) ALP mRNA expression levels. (**B**) Runx2 mRNA expression levels. (**C**) OCN mRNA expression levels. *P < 0.05, **P < 0.01, ***P < 0.001

ALP, Runx2, and OCN mRNA levels were significantly lower in the LPS, G-CSF, and G-CSF + LPS groups than in the control group (Fig. 4A-C, P < 0.05). In particular, pairwise comparisons between groups revealed a significant reduction in the G-CSF + LPS group (Fig. 4A-C).

### Effect of G-CSF on the protein expression levels of hPDLSC-associated osteogenic differentiation pathway components in the LPS-induced inflammatory microenvironment

**Fig. 5:**
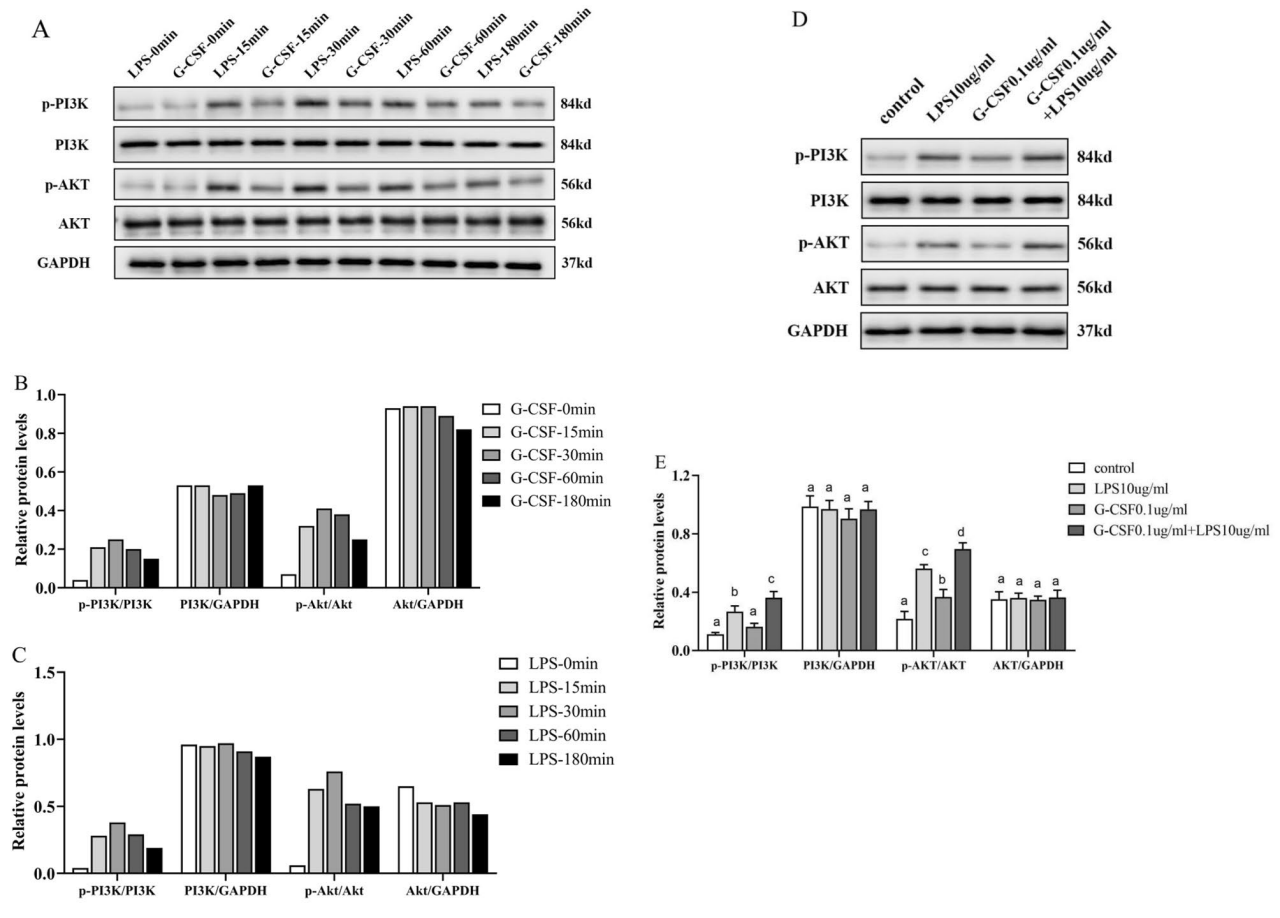
Western blotting revealed the protein expression levels of hPDLSC-associated osteogenic differentiation pathway components. (**A**–**C**) Protein expression levels of hPDLSC-associated osteogenic differentiation pathway components over time. (**D**, **E**) Protein expression levels of hPDLSC-associated osteogenic differentiation pathway components at 30 min. Pairwise comparisons were performed between different concentrations at the same time point; significant differences (*P* < 0.05) are indicated by different letters, while similar letters indicate no difference

The proteins expression levels of PI3K/Akt osteogenic differentiation pathway components were determined by Western blotting. Over the time course from 0 min to 3 h, the protein expression levels at 30 min were significantly higher than at other time points (Fig. 5A); thus, 30 min was selected as the time point for subsequent analysis. Western blotting revealed that p-PI3K and p-Akt levels were significantly higher in the G-CSF (Fig. 5B) and LPS groups (Fig. 5C) than in the control group (*P* < 0.05); however, there were no differences in the total protein levels of PI3K and Akt. Thus, G-CSF treatment during LPS-induced inflammation led to significantly enhanced expression of the hPDLSC-associated osteogenic differentiation pathway proteins p-PI3K and p-Akt, while it did not influence the total protein levels of PI3K or Akt (Fig. 5D-E, P < 0.05).

## Discussion

Periodontitis is one of the most common chronic infectious diseases in humans; this major oral disease causes tooth loss and endangers systemic health. The pathogenesis of periodontitis is not fully understood; thus, there is a lack of effective treatment for periodontitis. Although periodontitis is related to the activities of microorganisms in dental plaque, its progression and the resulting tissue destruction are mainly caused by an excessive host immune response which is mainly orchestrated by the upregulation of proinflammatory cytokines and related host inflammatory mediators [4, 27]. During the last decades, several inflammatory mediators have been revealed to be involved in the pathogenesis of periodontitis. Previous reports have demonstrated that, a proportional increase of salivary IL-6 was associated with a significant trend of periodontitis [28]. MMP-9 was associated with periodontal tissue damage during active stages of periodontitis [29]. Biomarkers were identified for better predict the onset and progression of periodontitis, Nod-like receptor family pyrin domain-containing protein-3 was assumed as a promising biomarker of disease risk in patients with periodontitis [30]. Also, G-CSF is an important immunomodulator that can specifically bind to its receptor to regulate multiple immune cell inflammatory responses, leading to the induction of G-CSF bioactivity [31]. G-CSFR is expressed throughout neutrophil differentiation and maturation; it is highly expressed in mature neutrophils. Various other immune cells (e.g., monocytes and macrophages) also express G-CSFR [31].

To our knowledge, this study demonstrated the expression of G-CSFR on hPDLSCs. Immunofluorescence analysis showed that G-CSFR was mainly localized at the cell membrane and in the cytoplasm. G-CSFR was initially found in various hematopoietic cells and non-hematopoietic tissues, including endothelial cells, cardiomyocytes, neural stem cells, and placental tissue cells [32]. Thus, in addition to the important roles of G-CSF in hematopoietic cell regulation and mobilization, it may have other functions in non-hematopoietic cells. For example, G-CSF increases adhesion receptor expression, while inducing endothelial cell proliferation and migration [32]. G-CSF signaling also promotes cardiomyocyte survival, stimulates neurogenesis, and enhances glioma cell migration; Moreover, G-CSF inhibits tumor cell migration and invasion [32]. Therefore, we hypothesized that in periodontal tissues, G-CSF would specifically bind to its receptor on hPDLSCs, thus initiating hPDLSC signaling and altering biological activity in those cells.

To characterize the effect of G-CSF on hPDLSC proliferation in the LPS-induced inflammatory microenvironment, we performed concentration gradient experiments. We found that the G-CSF treatment suppressed hPDLSC proliferation. Although the G-CSF + LPS group enhanced the inhibition of hPDLSC, this enhancement was not statistically significant. Specifically, When hPDLSCs were stimulated with ≥ 10 LPS µg/mL for 48 h, hPDLSC proliferation was inhibited; an LPS concentration of ≤ 1 µg/mL was able to promote hPDLSC proliferation. These findings suggested that hPDLSC proliferation is promoted during early stages of inflammation, when hPDLSCs showed greater mitotic activity. When hPDLSCs were stimulated with LPS for 72 h, the inflammatory microenvironment gradually suppressed hPDLSC proliferation. Therefore, we selected an LPS concentration of 10 µg/mL at 48 h for our in vitro inflammatory microenvironment. Consistent with our results, Kato reported that 1 µg/mL and 10 µg/mL LPS affected hPDLSC osteogenic differentiation and upregulated the production of interleukins 1, 6 and 8; thus, 10 µg/mL LPS was regarded as the optimal concentration to induce an inflammatory microenvironment during analysis of hPDLSCs [33]. Similarly, as the concentration of G-CSF increased, prolonged stimulation led to the inhibition of hPDLSC proliferation; this inhibitory effect gradually increased as the concentration of G-CSF increased. Accordingly, the moderate cytoinhibitory concentration of 0.1 µg/mL G-CSF for 48 h was used for subsequent experiments. LPS is one of the main pathogenic substances of gram-negative bacteria; it is regarded as a key factor in the occurrence and progression of periodontitis [34]. In vitro cell culture experiments showed that LPS from *P. gingivalis* was able to promote a nearly 15-fold increase in G-CSF gene expression levels in human gingival fibroblasts [13]. The G-CSF gene expression level increased by 36-fold in a three-dimensional co-culture model of gingival epithelial cells and fibroblasts during stimulation with LPS from *Aggregatibacter actinomycetemcomitans* [9]. Thus, many in vitro cell culture experiments have confirmed that G-CSF gene expression is promoted by infection with periodontal pathogenic bacteria. The above evidences suggested that the periodontal inflammatory environment causes a local increase in G-CSF and suppresses hPDLSC activity.

Subsequently, we explored the effects of G-CSF on hPDLSC osteogenic differentiation capacity and expression patterns of osteogenesis-related genes. The results indicated that G-CSF inhibited hPDLSC osteogenic differentiation capacity and reduced the expression levels of osteogenesis-related genes in the LPS-induced inflammatory microenvironment at 48 h; these changes may be influenced alveolar bone repair and regeneration. G-CSF induced hPDLSCs after osteogenesis for 21 days in the inflammatory microenvironment induced by LPS. The control group exhibited a large area of orange-red calcium nodules, while the LPS group exhibited a small area of nodules (Fig. 3B); the area was greatly decreased in the G-CSF group (Fig. 3C) and nearly absent in the G-CSF + LPS group. Osteogenic differentiation is an important property of stem cells; after exposure to a specific induction medium, stem cells can differentiate into osteoblasts, with calcium salt deposits on the cell surface that eventually form orange-red calcium nodules [35]. The expression patterns of osteogenesis-related genes in hPDLSCs were examined by RT-PCR at 48 h. In the present study, G-CSF and LPS both reduced the mRNA expression levels of ALP, Runx2, and OCN of hPDLSCs; Furthermore, G-CSF + LPS group caused significant reduction of OCN mRNA than other groups, while no statistically significant difference in ALP and Runx2 expression between the G-CSF + LPS group and LPS group. Studies have highlighted that OCN was used to identify osteoblast-specific transcription factors and to define molecular bases of bone physiology [36]. In our study, we selected 48 h to assumed the changes of osteogenic capacity, which may be resulted in the discrepancy. G-CSF is known to block proliferation and osteogenic differentiation in hematopoietic stem cells, induce apoptosis in osteoblasts, and reduce new bone formation [25]. After G-CSF induction enhanced the mobilization of hematopoietic stem cells, osteoblast number and function both decreased, while OCN expression significantly decreased [37]. The mobilization of G-CSF is associated osteoblast inhibition [38], accompanied by increased osteoblast apoptosis [18] and osteoblast flattening [38], with significantly reduced expression levels of many immune system components. Experiments in a mouse model revealed that G-CSF significantly downregulated the expression of Runx2, induced by Staphylococcus aureus infection in mice [38].

Western blotting analysis showed that G-CSF increased the protein expression levels of hPDLSC pathway components p-PI3K and p-Akt in the LPS-induced inflammatory microenvironment, suggesting that G-CSF inhibited hPDLSC activity may be by activating the PI3K/Akt signaling pathway. G-CSF reportedly activates metalloproteinase-2 through the PI3K/Akt pathway [39]. The PI3K/Akt pathway is associated with multiple cellular functions, including survival, proliferation, differentiation, angiogenesis, migration, and invasion [40]; activation of this pathway leads to the phosphorylation of several key downstream targets [40]. G-CSF has been shown to inhibit GSK-3 through the PI3K/Akt pathway, thereby alleviating inflammation in nerve tissue [41]. The PI3K/Akt pathway is involved in regulating the mRNA expression of Toll-liked receptor 4, as well as the response to LPS [42]. Moreover, OCN-associated cell proliferation is controlled by the PI3K/Akt signaling pathway [43].

Our results suggested that G-CSF may be an important inflammatory factor that mediated hPDLSC proliferation and osteogenic differentiation, possibly by activating the PI3K/Akt signaling pathway, although the specific mechanism remains unclear. Further studies are needed to determine the specific signaling mechanisms by which G-CSF affects hPDLSCs.

## Conclusions

This study demonstrated that G-CSFR was expressed on hPDLSCs. Moreover, G-CSF significantly inhibited hPDLSC proliferation. Also, in the LPS-induced inflammatory microenvironment, G-CSF inhibited hPDLSC osteogenic differentiation and reduced the expression levels of osteogenesis-related genes. G-CSF increased the protein expression levels of hPDLSC pathway components p-PI3K and p-Akt, but the specific mechanism of PI3K/Akt signaling pathway remains further studies by which G-CSF affected hPDLSCs. These findings indicated that G-CSF may be one of the essential immune factors that mediated the activity of hPDLSC. These insights may help to improve the osteogenic differentiation of hPDLSCs for use in periodontal repair and regeneration in novel immune-mediated treatments.

## Electronic supplementary material

Below is the link to the electronic supplementary material.

**Additional File 1: Fig. S1.** Characteristics of human periodontal ligament stem cells (hPDLSCs). Bar: 100 ?m. **Fig. S2.** Immunofluorescence identification of hPDLSCs. Green immunofluorescence represents STRO-1 (A). DAPI-stained blue fluorescence represents nuclei (B). Bar: 50 ?m. **Fig. S3.** Genotyping results and typing profiles of short tandem repeats and amelogeninloci.

**Additional File 2:** Original images of all blots.

## Data Availability

All raw data used and analyzed during the current research are available from the corresponding author on reasonable request.

## List of abbreviations

G-CSF: Granulocyte colony-stimulating factor
hPDLSCs: Human periodontal ligament stem cells
G-CSFR: Granulocyte colony-stimulating factor receptor
LPS: lipopolysaccharide
CCK-8: Cell-Counting Kit 8
ALP: alkaline phosphatase
Runx2: runt-related transcription factor 2
OCN: osteocalcin
PI3K: phosphatidylinositol 3-kinase
Akt: protein kinase B
RT-PCR: Reverse transcription-polymerase chain reaction
GAPDH: glyceraldehyde-3-phosphate dehydrogenase
