# Supplementary material for: Effects of G-CSF on hPDLSC proliferation and osteogenic differentiation in the LPS-induced inflammatory microenvironment
Source: BMC Oral Health. 2023 Jun 26;23:422. doi: 10.1186/s12903-023-03040-9 (PMC10294445; doi:10.1186/s12903-023-03040-9)
Supplement: Supplementary file 1 — Additional File 1: Fig. S1. Characteristics of human periodontal ligament stem cells (hPDLSCs). Bar: 100 ?m. Fig. S2. Immunofluorescence identification of hPDLSCs. Green immunofluorescence represents STRO-1 (A). DAPI-stained blue fluorescence represents nuclei (B). Bar: 50 ?m. Fig. S3. Genotyping results and typing profiles of short tandem repeats and amelogeninloci. [file 12903_2023_3040_MOESM1_ESM.docx]

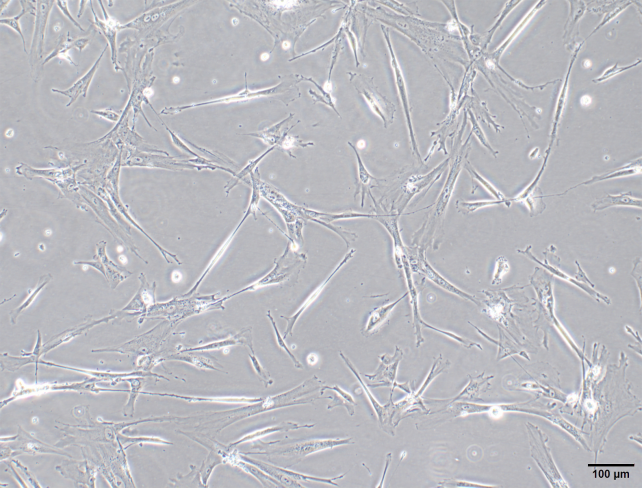


**Fig S1** Characteristics of human periodontal ligament stem cells (hPDLSCs). Bar: 100 µm.


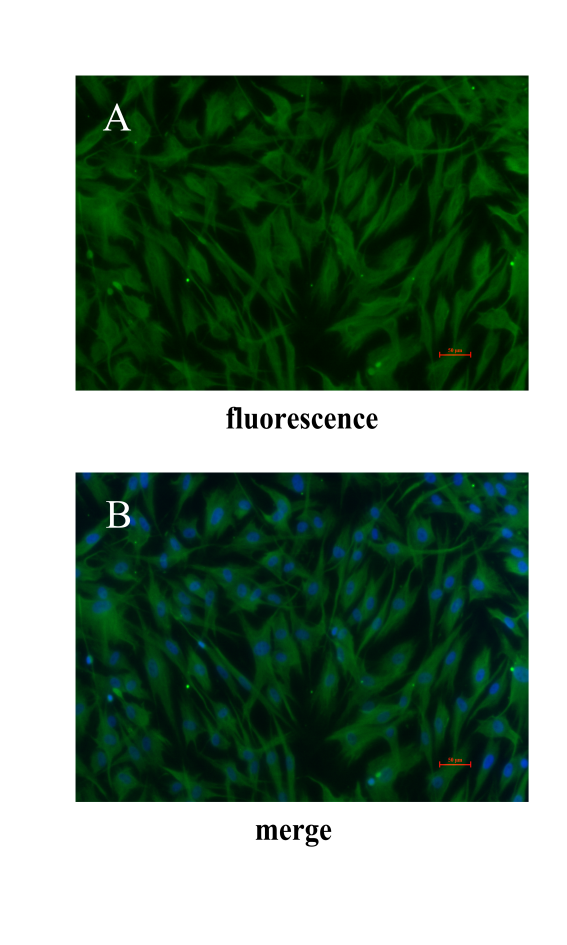


**Fig S2** Immunofluorescence identification of hPDLSCs. Green immunofluorescence represents STRO-1 (A). DAPI-stained blue fluorescence represents nuclei (B). Bar: 50 µm.


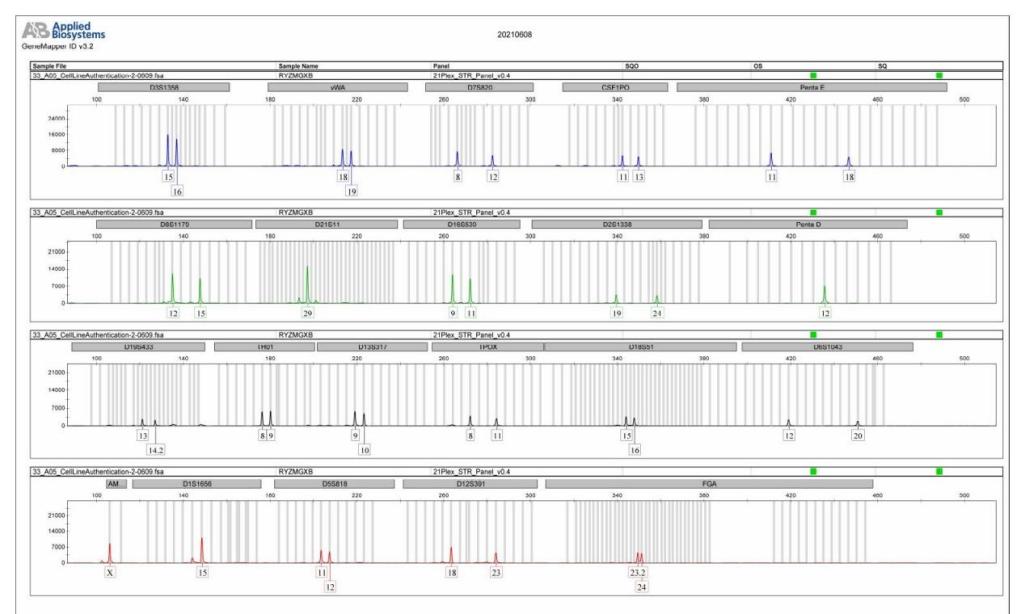


**Fig S3** Genotyping results and typing profiles of short tandem repeats and amelogeninloci.
