# Supplementary material for: Effects of G-CSF on hPDLSC proliferation and osteogenic differentiation in the LPS-induced inflammatory microenvironment
Source: BMC Oral Health. 2023 Jun 26;23:422. doi: 10.1186/s12903-023-03040-9 (PMC10294445; doi:10.1186/s12903-023-03040-9)
Supplement: Supplementary file 2 — Additional File 2: Original images of all blots. [file 12903_2023_3040_MOESM2_ESM.pptx]

## Slide 1
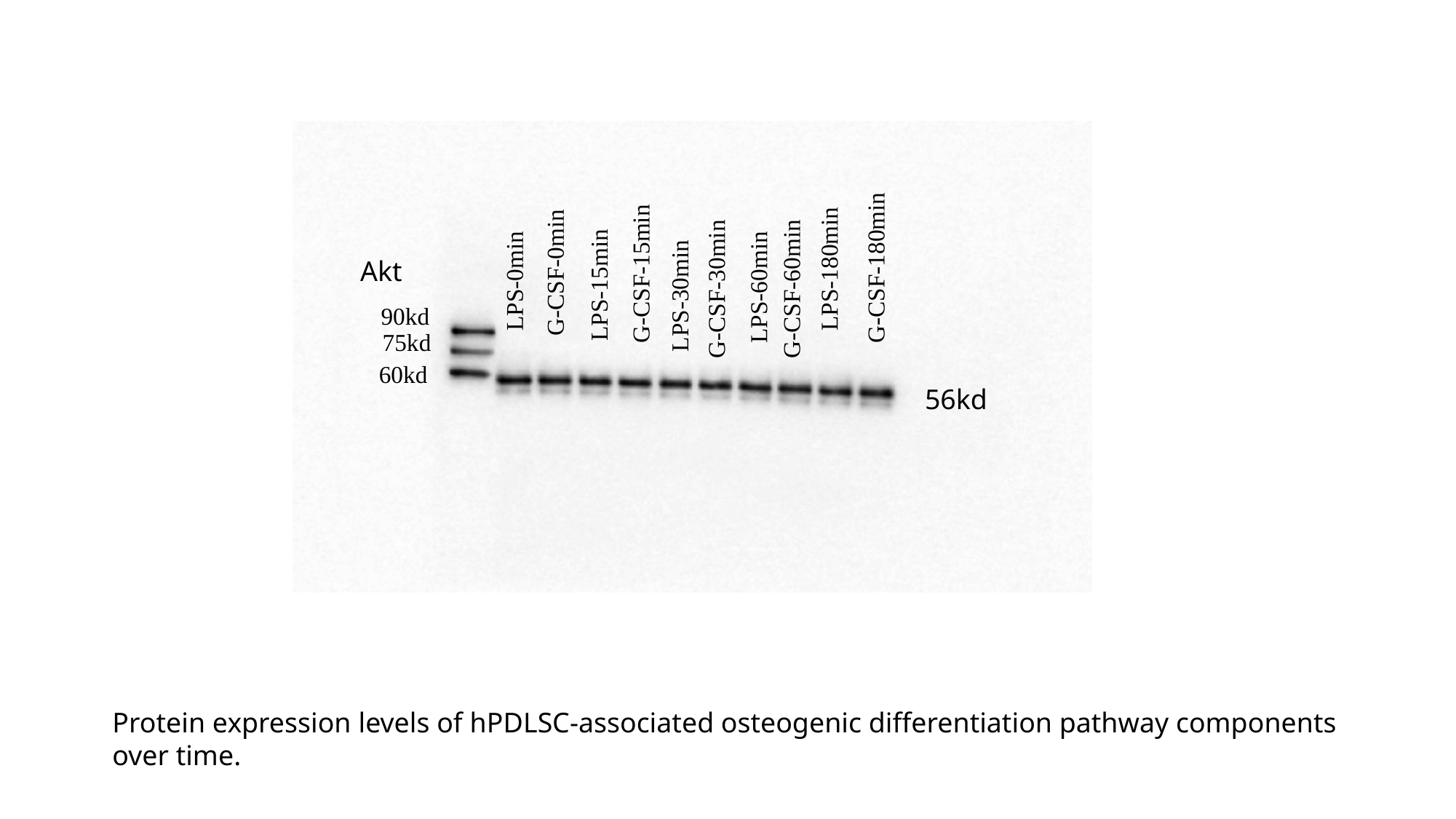

LPS-0min
G-CSF-180min
LPS-180min
G-CSF-15min
Akt
G-CSF-0min
LPS-15min
LPS-60min
G-CSF-60min
G-CSF-30min
LPS-30min
90kd
75kd
60kd
56kd
Protein expression levels of hPDLSC-associated osteogenic differentiation pathway components over time.

## Slide 2
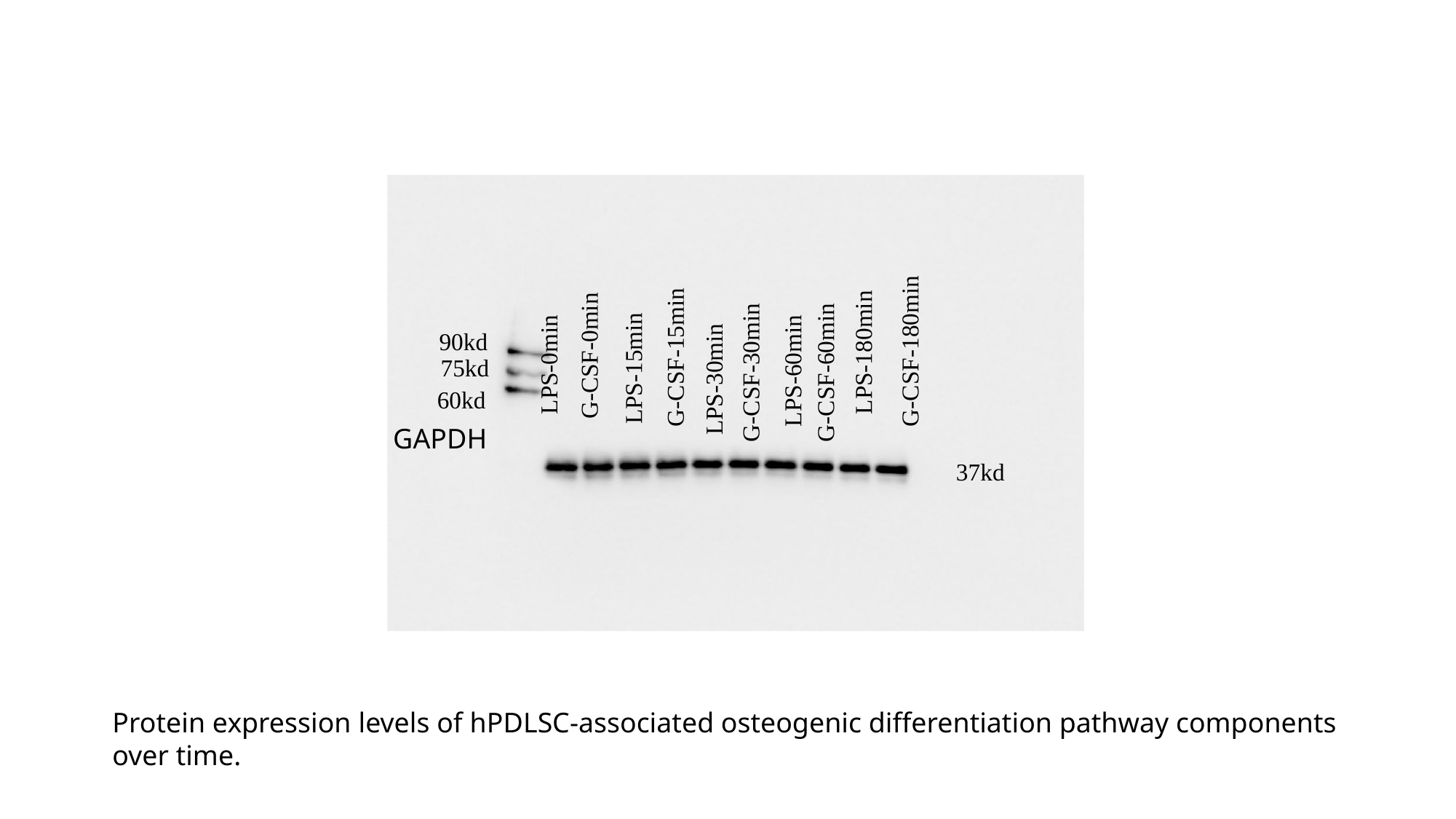

LPS-0min
90kd
G-CSF-180min
LPS-180min
G-CSF-15min
G-CSF-0min
LPS-15min
LPS-60min
G-CSF-60min
G-CSF-30min
75kd
LPS-30min
60kd
GAPDH
37kd
Protein expression levels of hPDLSC-associated osteogenic differentiation pathway components over time.

## Slide 3
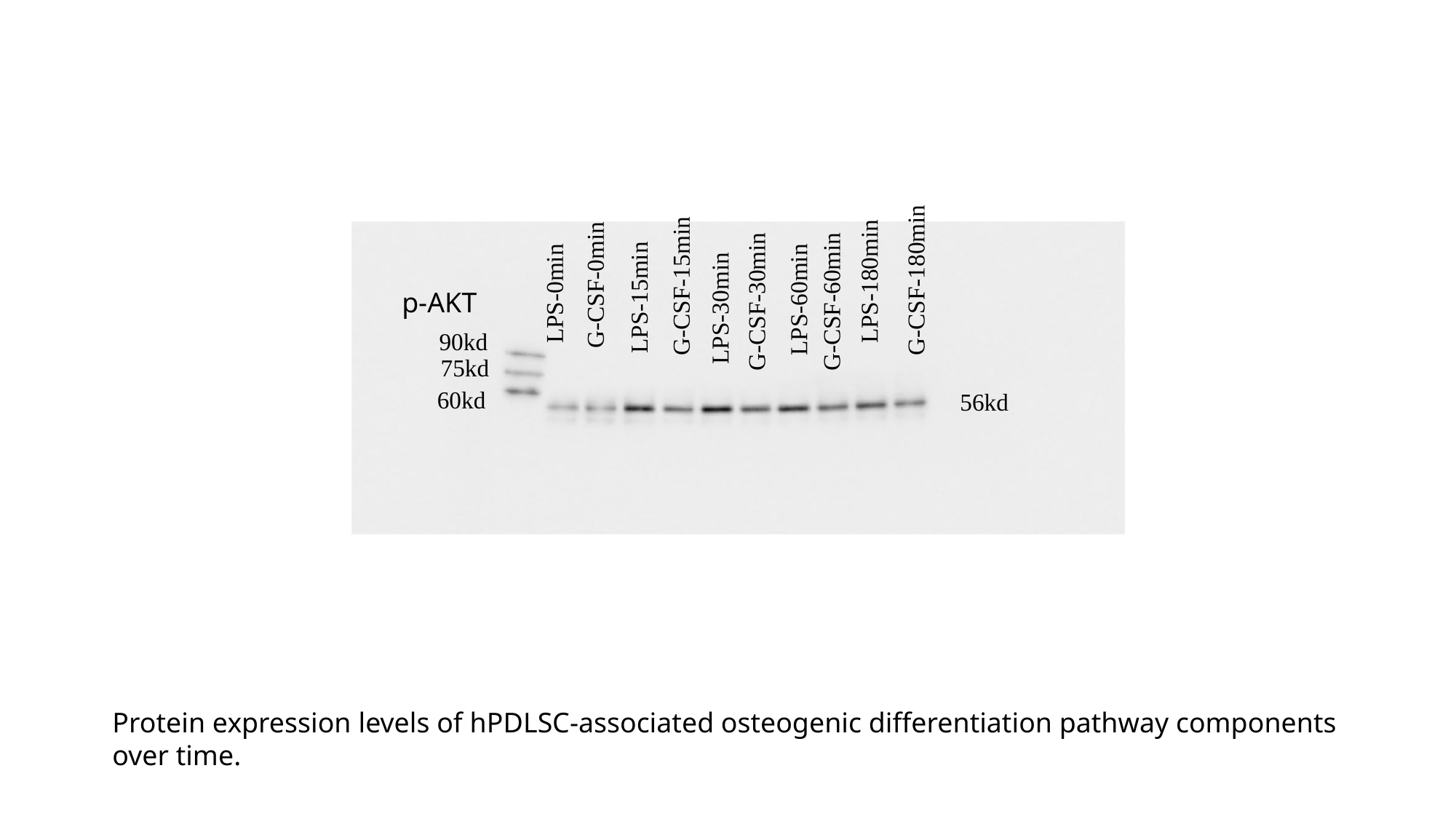

LPS-0min
G-CSF-180min
LPS-180min
G-CSF-15min
G-CSF-0min
LPS-15min
LPS-60min
G-CSF-60min
G-CSF-30min
p-AKT
LPS-30min
90kd
75kd
60kd
56kd
Protein expression levels of hPDLSC-associated osteogenic differentiation pathway components over time.

## Slide 4
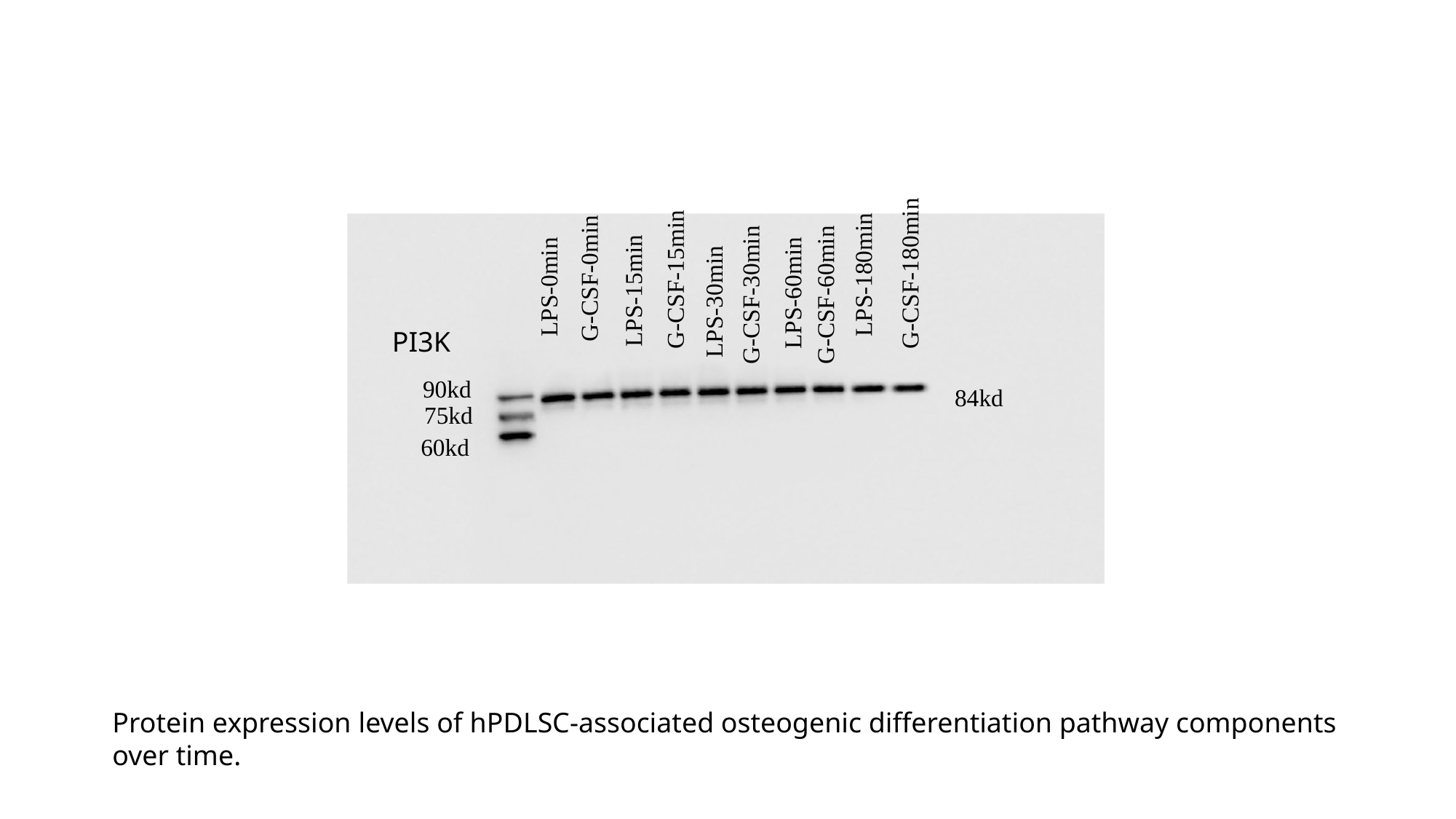

LPS-0min
G-CSF-180min
LPS-180min
G-CSF-15min
G-CSF-0min
LPS-15min
LPS-60min
G-CSF-60min
G-CSF-30min
LPS-30min
PI3K
90kd
84kd
75kd
60kd
Protein expression levels of hPDLSC-associated osteogenic differentiation pathway components over time.

## Slide 5
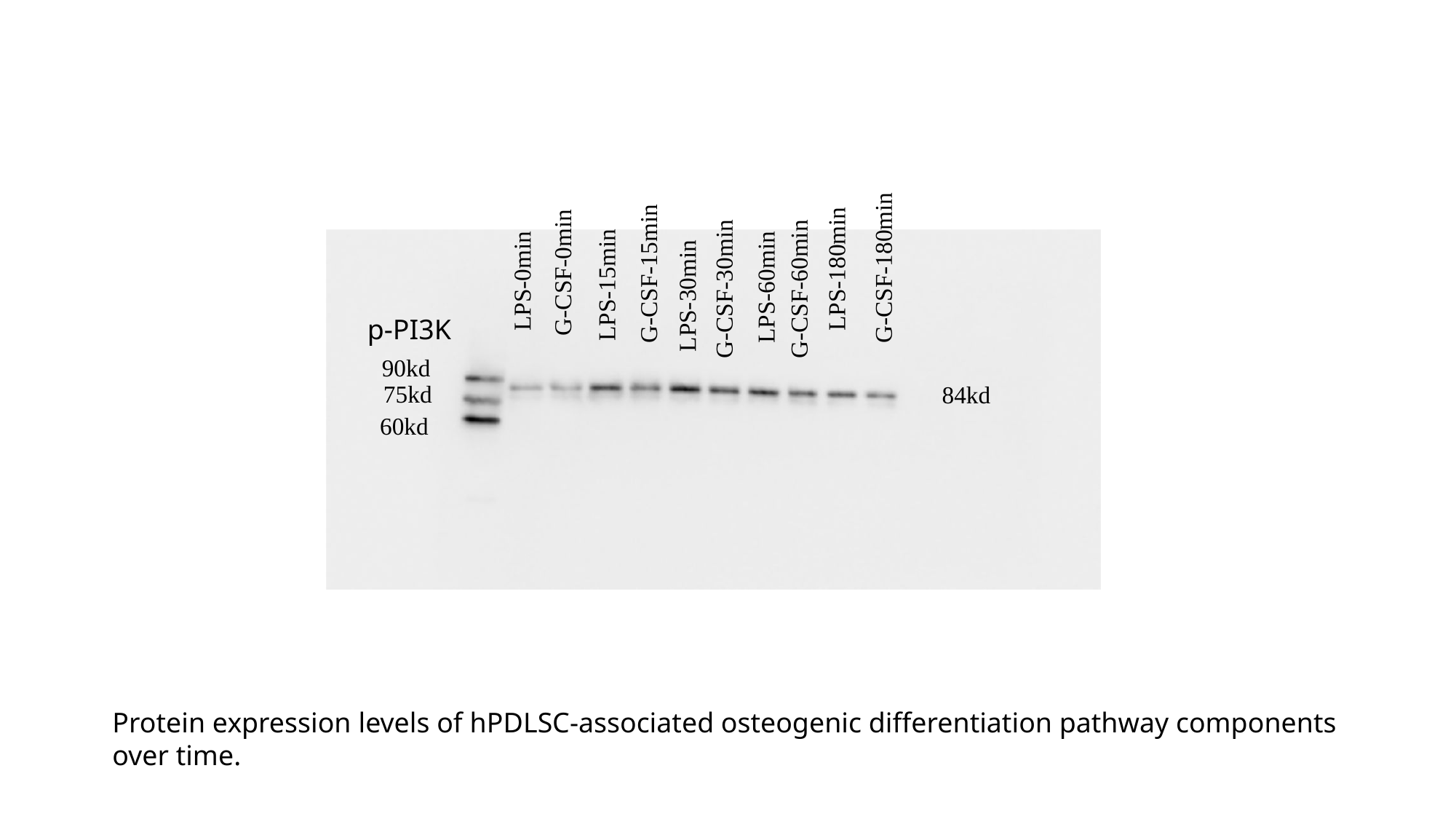

LPS-0min
G-CSF-180min
LPS-180min
G-CSF-15min
G-CSF-0min
LPS-15min
LPS-60min
G-CSF-60min
G-CSF-30min
LPS-30min
p-PI3K
90kd
75kd
84kd
60kd
Protein expression levels of hPDLSC-associated osteogenic differentiation pathway components over time.

## Slide 6
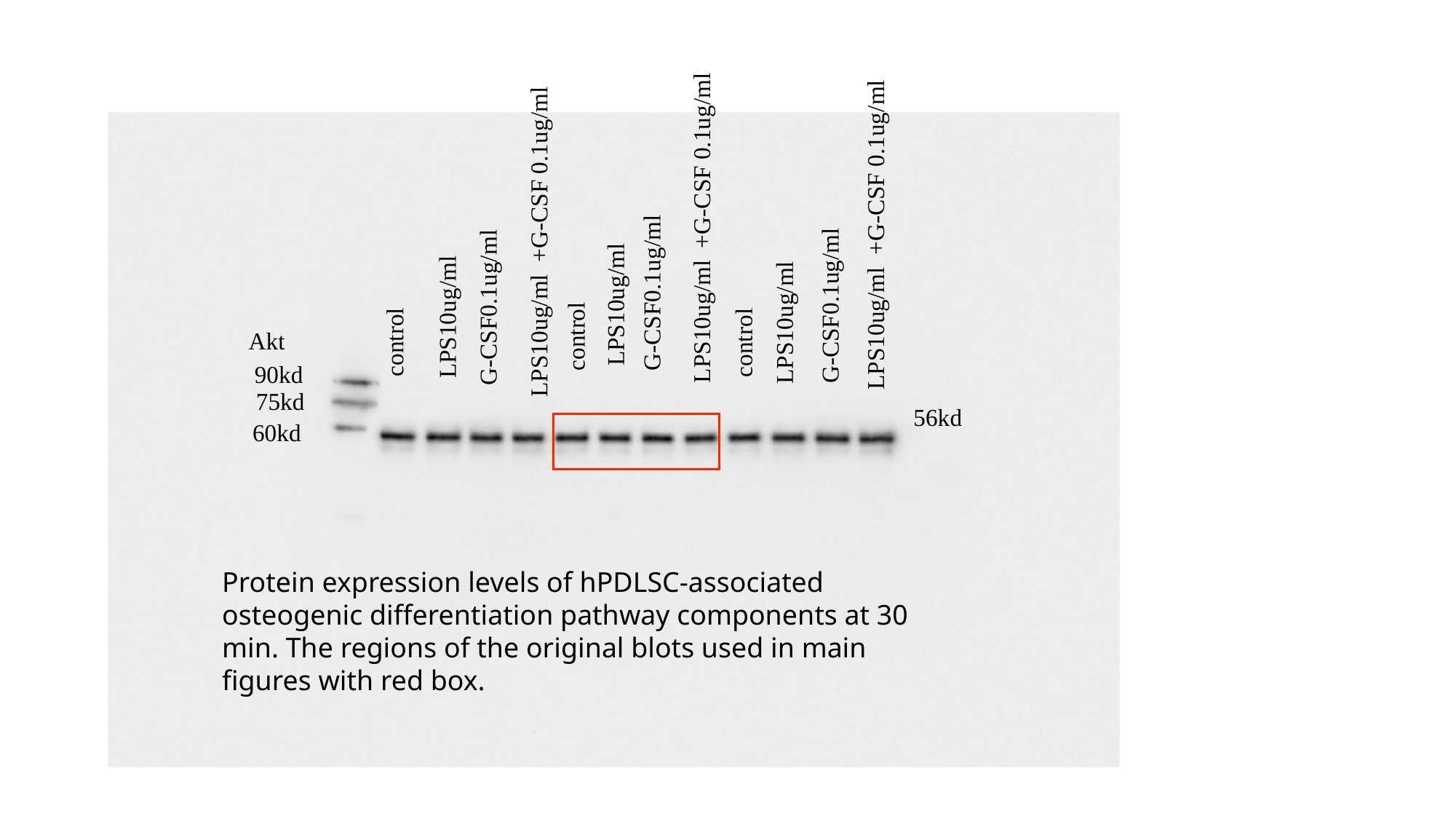

LPS10ug/ml +G-CSF 0.1ug/ml
LPS10ug/ml +G-CSF 0.1ug/ml
LPS10ug/ml +G-CSF 0.1ug/ml
G-CSF0.1ug/ml
LPS10ug/ml
G-CSF0.1ug/ml
G-CSF0.1ug/ml
LPS10ug/ml
LPS10ug/ml
control
Akt
control
control
90kd
75kd
56kd
60kd
Protein expression levels of hPDLSC-associated osteogenic differentiation pathway components at 30 min. The regions of the original blots used in main figures with red box.

## Slide 7
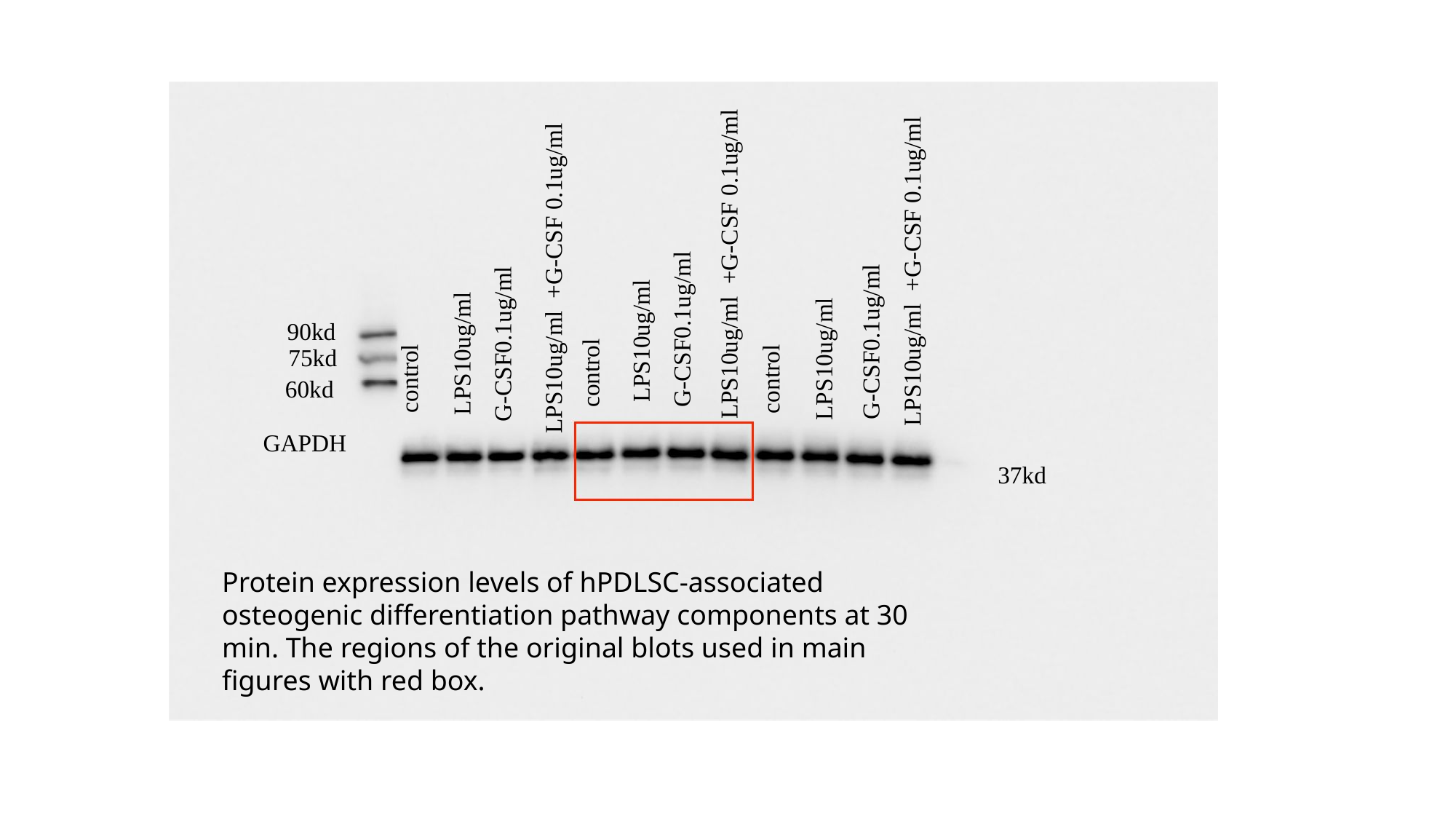

LPS10ug/ml +G-CSF 0.1ug/ml
LPS10ug/ml +G-CSF 0.1ug/ml
LPS10ug/ml +G-CSF 0.1ug/ml
G-CSF0.1ug/ml
90kd
LPS10ug/ml
G-CSF0.1ug/ml
G-CSF0.1ug/ml
LPS10ug/ml
75kd
LPS10ug/ml
control
control
control
60kd
GAPDH
37kd
Protein expression levels of hPDLSC-associated osteogenic differentiation pathway components at 30 min. The regions of the original blots used in main figures with red box.

## Slide 8
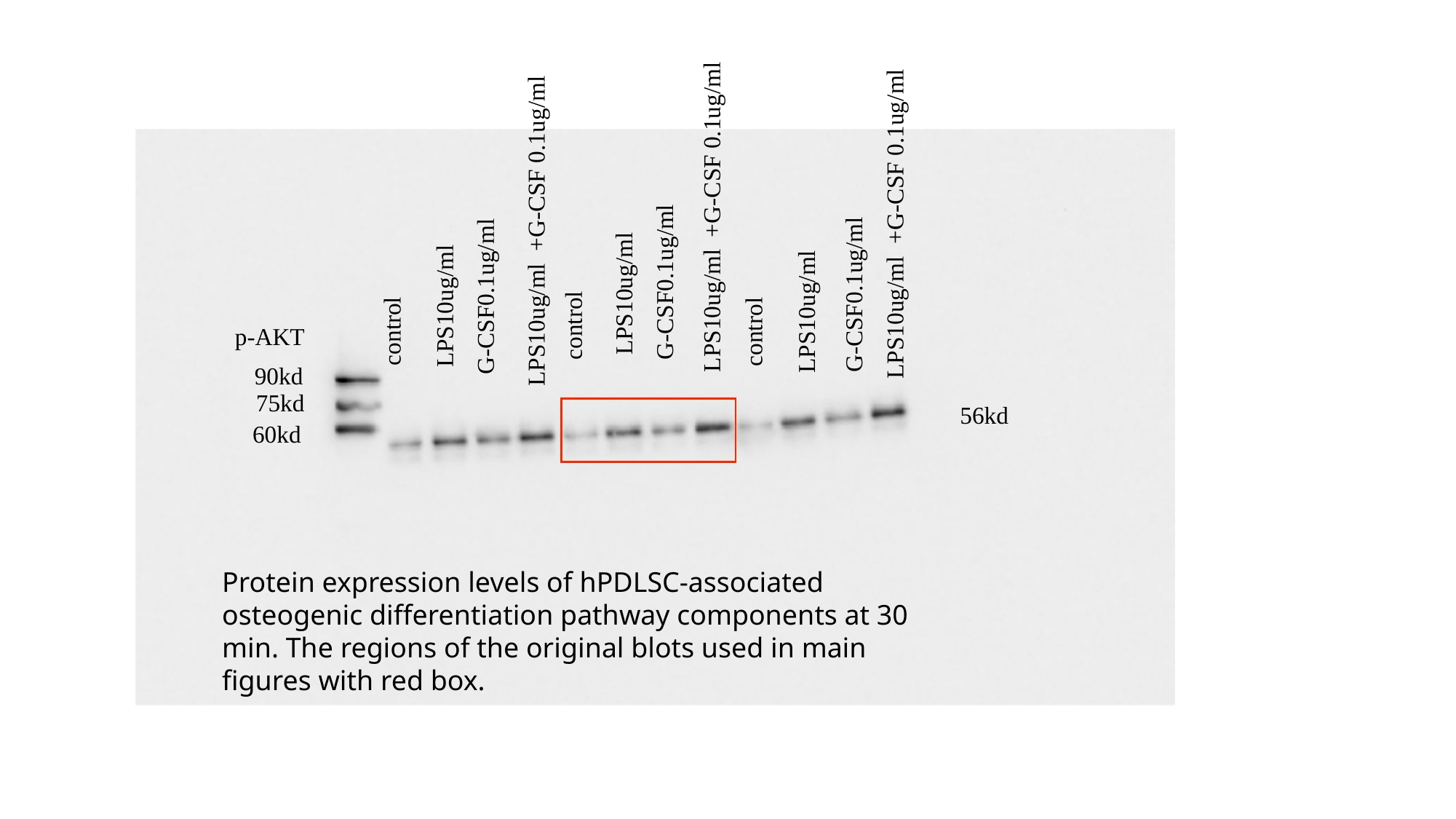

LPS10ug/ml +G-CSF 0.1ug/ml
LPS10ug/ml +G-CSF 0.1ug/ml
LPS10ug/ml +G-CSF 0.1ug/ml
G-CSF0.1ug/ml
LPS10ug/ml
G-CSF0.1ug/ml
G-CSF0.1ug/ml
LPS10ug/ml
LPS10ug/ml
control
control
control
p-AKT
90kd
75kd
56kd
60kd
Protein expression levels of hPDLSC-associated osteogenic differentiation pathway components at 30 min. The regions of the original blots used in main figures with red box.

## Slide 9
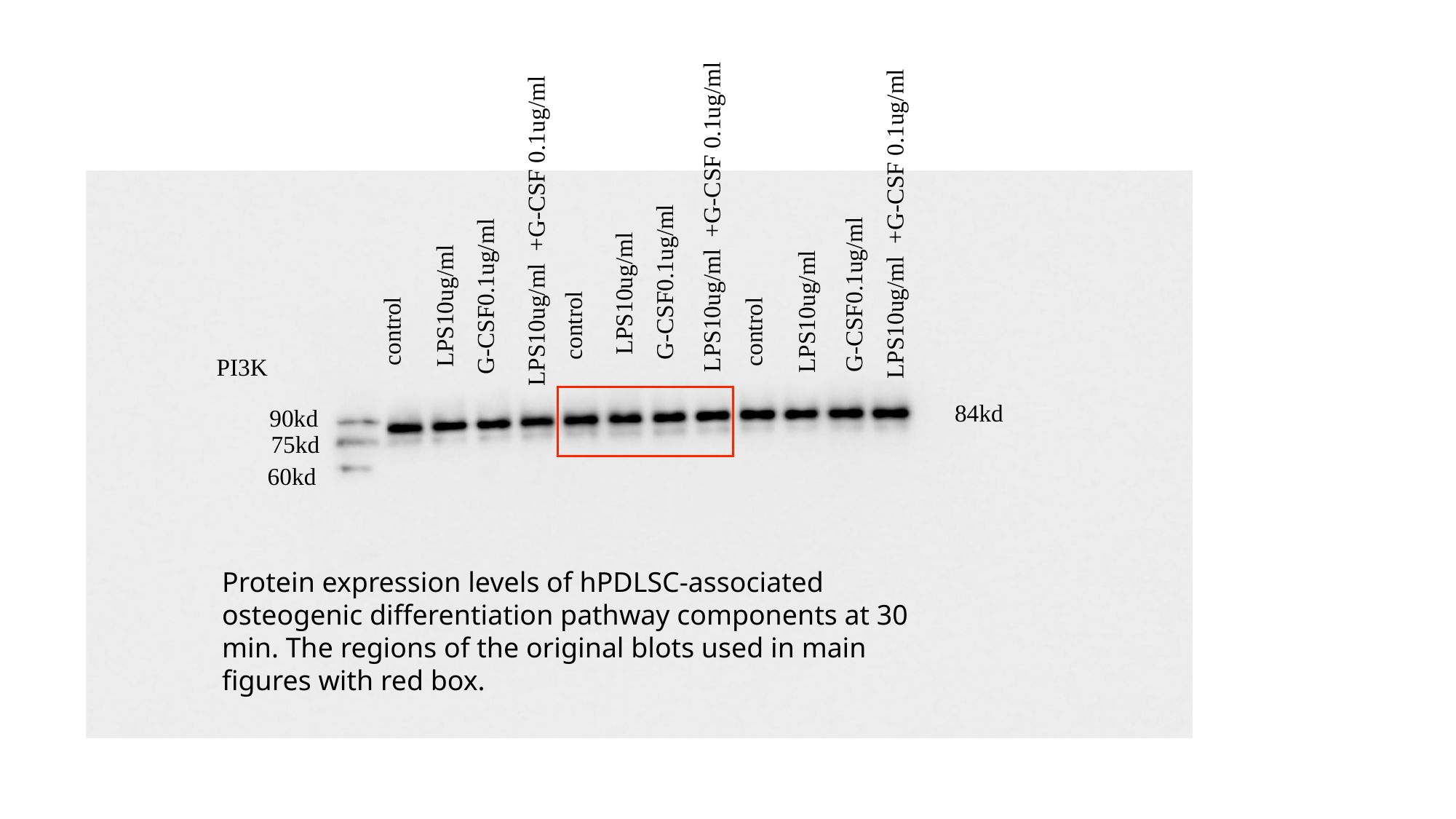

LPS10ug/ml +G-CSF 0.1ug/ml
LPS10ug/ml +G-CSF 0.1ug/ml
LPS10ug/ml +G-CSF 0.1ug/ml
G-CSF0.1ug/ml
LPS10ug/ml
G-CSF0.1ug/ml
G-CSF0.1ug/ml
LPS10ug/ml
LPS10ug/ml
control
control
control
PI3K
84kd
90kd
75kd
60kd
Protein expression levels of hPDLSC-associated osteogenic differentiation pathway components at 30 min. The regions of the original blots used in main figures with red box.

## Slide 10
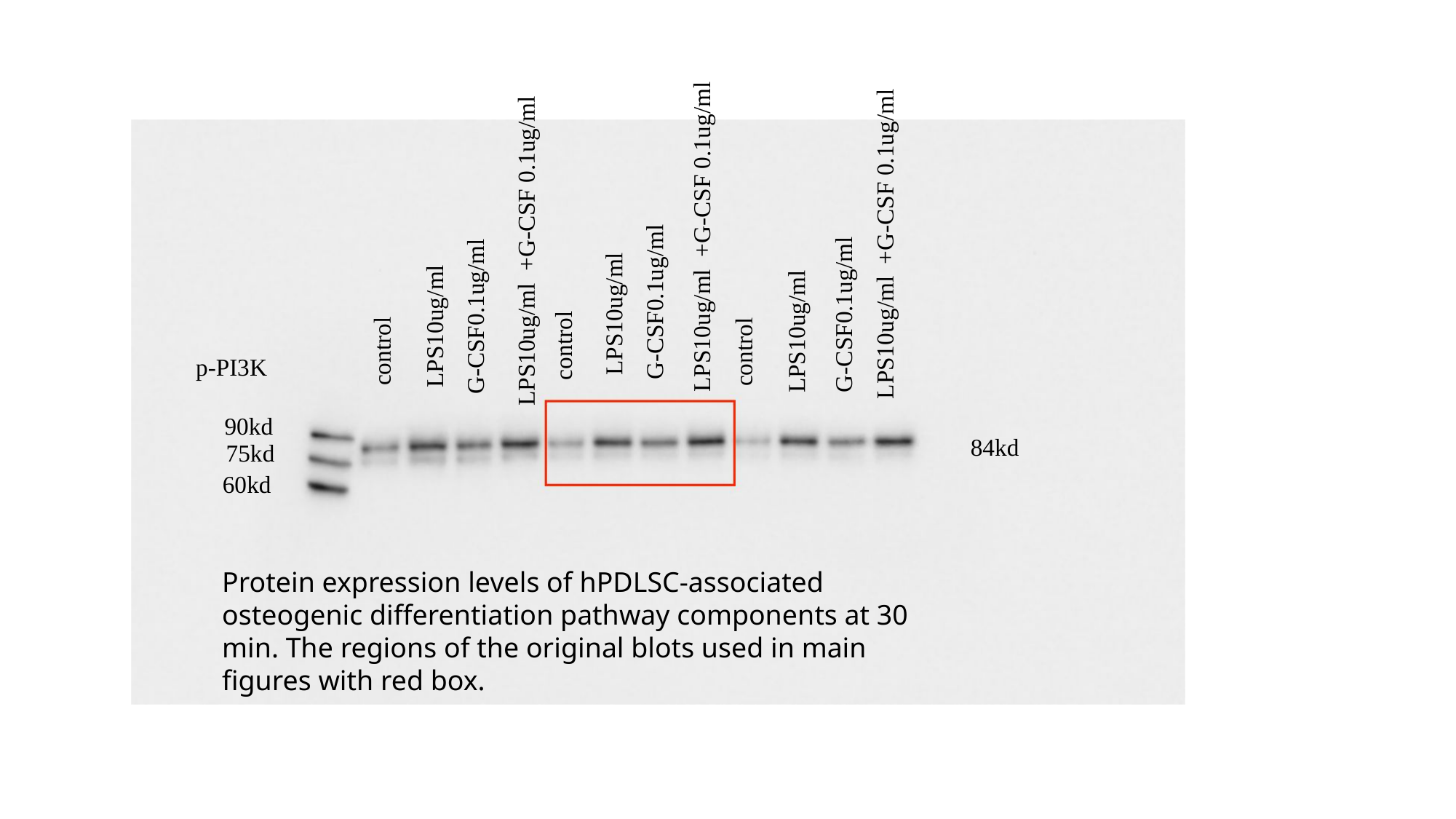

LPS10ug/ml +G-CSF 0.1ug/ml
LPS10ug/ml +G-CSF 0.1ug/ml
LPS10ug/ml +G-CSF 0.1ug/ml
G-CSF0.1ug/ml
LPS10ug/ml
G-CSF0.1ug/ml
G-CSF0.1ug/ml
LPS10ug/ml
LPS10ug/ml
control
control
control
p-PI3K
90kd
84kd
75kd
60kd
Protein expression levels of hPDLSC-associated osteogenic differentiation pathway components at 30 min. The regions of the original blots used in main figures with red box.
